# Deep hierarchical embedding for simultaneous modeling of GPCR proteins in a unified metric space

**DOI:** 10.1038/s41598-021-88623-8

**Published:** 2021-05-05

**Authors:** Taeheon Lee, Sangseon Lee, Minji Kang, Sun Kim

**Affiliations:** 1 Looxid Labs, Seoul, 06628 Republic of Korea; 2 BK21 FOUR Intelligence Computing, Seoul National University, Seoul, 08826 Republic of Korea; 3 Department of Computer Science, Stanford University, Stanford, CA 94305 USA; 4 Bioinformatics Institute, Seoul National University, Seoul, 08826 Republic of Korea; 5 Department of Computer Science and Engineering, Seoul National University, Seoul, 08826 Republic of Korea; 6 Interdisciplinary Program in Bioinformatics, Seoul National University, Seoul, 08826 Republic of Korea; 7 Institute of Engineering Research, Seoul National University, Seoul, 08826 Republic of Korea

**Keywords:** Bioinformatics, Computational models, Phylogeny

## Abstract

GPCR proteins belong to diverse families of proteins that are defined at multiple hierarchical levels. Inspecting relationships between GPCR proteins on the hierarchical structure is important, since characteristics of the protein can be inferred from proteins in similar hierarchical information. However, modeling of GPCR families has been performed separately for each of the family, subfamily, and sub-subfamily level. Relationships between GPCR proteins are ignored in these approaches as they process the information in the proteins with several disconnected models. In this study, we propose DeepHier, a deep learning model to simultaneously learn representations of GPCR family hierarchy from the protein sequences with a unified single model. Novel loss term based on metric learning is introduced to incorporate hierarchical relations between proteins. We tested our approach using a public GPCR sequence dataset. Metric distances in the deep feature space corresponded to the hierarchical family relation between GPCR proteins. Furthermore, we demonstrated that further downstream tasks, like phylogenetic reconstruction and motif discovery, are feasible in the constructed embedding space. These results show that hierarchical relations between sequences were successfully captured in both of technical and biological aspects.

## Introduction

G protein-coupled receptor (GPCR) is the largest transmembrane protein family and one of the most extensively investigated drug targets^1,2^. Recently, unexplored GPCR proteins are investigated as novel drug targets for several diseases^3^. Furthermore, a recent study demonstrated that understanding genetic variations in GPCR proteins could enhance the effectiveness of drugs^4^. Thus, precise characterization of GPCR families can help accelerate drug discovery. GPCR is classified in a hierarchical class structure, represented by family, subfamily, and sub-subfamily level classes. This structure was constructed following the sequence similarity and evolutionary histories of the proteins^5^. Analyzing the characteristics of the proteins with respect to this structure lies at the heart of GPCR studies^6^ and inspecting the relations between GPCR sequences regarding this structure is an important research subject for two main reasons. First, properties of the protein are often inferred from the existing proteins that have already been experimentally validated^7^. Second, evolutionary history of the proteins can be revealed from the relations among proteins.

Approaches based on machine learning techniques, such as hierarchical classification and clustering, on the GPCR class structure have been widely explored. For example, classifiers that are designed specifically at each hierarchical family level were introduced to classify GPCR proteins. In this approach, classification was proceeded in a top-down manner from family classes to sub-subfamily classes^8,9^. Unsupervised clustering algorithm was also proposed to investigate the properties of GPCR proteins like sequence similarity, classification of GPCR sequences, and phylogenetic tree reconstruction^6^. These methods have shown successes in modeling GPCR fairly accurately. However, existing methods had to model GPCR at each of the family hierarchies separately and inevitably employed series of separate steps to deal with the features in the class structure since the representations used in the methods can hardly reveal unified features across the class hierarchies. Otherwise, they had to compute the distances between protein sequences for all pairs, which is computationally too heavy^6,9,10^. As a result, processing complex features throughout the hierarchy levels as a whole is nearly impossible with previous representations^11^. In the sense that GPCR class hierarchy was constructed using complex features including phylogenetic traits, ligand types and their functions^12^, these approaches may not provide research opportunities to inspect the relations between these traits throughout the GPCR proteins. In this regard, it is important to construct comprehensive representations of GPCR proteins with hierarchical features inclusively incorporated.

In this work, we present DeepHier, a novel method that simultaneously learns and represents complex protein features across the hierarchies. Our end-to-end deep learning network constructs a single embedding space of GPCR sequences where hierarchical sequence information is preserved in terms of metric distances. DeepHier incorporates significant features across all hierarchical levels into a single vector. On the constructed embedding space, distances in the embedding space can be utilized in analyzing GPCR proteins for several downstream tasks.

In a series of experiments, we showed that the metric space generated by DeepHier successfully reflected hierarchical class structure of GPCR proteins. First, we extensively investigated distances among sequences in the embedding space using cluster analysis techniques. In the distance-based cluster analysis on the embedding space, proteins are grouped well according to the hierarchical class labels of proteins. Second, we showed that phylogenetic trees constructed from the embedding vectors accurately reflected the phylogeny of the proteins. In addition, we showed that biologically significant motifs in the GPCR proteins are well-represented in each cluster. We further investigated how those motifs are generalized or narrowed along different hierarchical levels. Last but not least, experiments on the query search demonstrated that similarity search on proteins can be done fairly efficiently with the embedding vectors.

## Related work

### Analyzing biological sequences with deep learning

Alignment-base algorithms achieved remarkable successes in identifying characteristics of biological sequences. However, performance of alignment-based algorithms might deteriorate significantly when there are large variations in sequences such as duplication or deletions of subsequences^13^. Furthermore, multiple sequence alignment algorithms require huge amount of computation time with respect to the number of sequences^14^. For these reasons, a number of alignment-free methods have been developed to achieve performances comparable to alignment-based algorithms.

Recently, deep learning technologies have brought unprecedented advances in analyzing biological sequences. For example, a previous deep learning study proposed deep convolutional layers to recognize protein folds from sequences with competitive accuracy and robustness^15^. Other works showed that biological sequences can be successfully modeled with a one-layer convolutional layer equipped with one-max pooling operator^16,17^. In these works, each convolutional filter was trained to acquire features that correspond to significant sequence patterns. Especially, DeepFam^17^ showed promising results in modeling proteins families with motif features. Recent deep learning work extended motif discovery components in DeepFam by adopting word2vec techniques to model distributional dependencies among nucleotides^18^. Although DeepHier is built upon the architecture proposed in DeepFam, it differs from the previous works in that neural network architecture is significantly expanded to incorporate hierarchical class information into a single model. Furthermore, our work enables the embedding of hierarchical features, which are originated from the protein family hierarchy, onto a single metric space.

### Metric learning on deep learning

With the success of deep learning models, more and more works have been proposed to utilize distances in the deep feature space. Training objective in these studies are to learn an effective mapping function of data where similarity between inputs can be directly inferred from the embedding vectors. Neural networks are trained to favor close distances between representations for data points of similar properties and distant representations for dissimilar data. For example, Siamese networks^19^ and Triplet networks^20^ were introduced in order to make feature vectors for similar data points to be located closely in distances in the proposed deep embedding spaces. Similarly, center loss was introduced to construct compact intra-class representations and separable inter-class representations^21^. In center loss, mean embedding vectors of data classes are employed as reference vectors to guide training. DeepHier adopted center loss to learn compact and separable embedding function according to protein family information.

In deep metric learning for sequence analyses, components of training phases are designed to grant biologically significant meanings to distances in the embedding space. For instances, a Siamese neural network for biological sequences was introduced, where alignment distances between sequences can be directly estimated from the embedding vectors of the given sequences^22^. Moreover, an embedding function based on long short-term memory was suggested to incorporate structural similarities of proteins during training^23^. While previous studies learned metrics for single level information of biological sequences, up to our knowledge, our work is a first work to model hierarchical relations between protein sequences into a single metric space.

## Methods

### Neural network architecture

The overall structure of DeepHier is extended and modified from DeepFam^17^. DeepFam is a one-dimensional convolutional neural network with different convolutional filter sizes that can model sequence motifs of various lengths. Due to the success of DeepFam in modeling protein families, this architecture has been employed as a baseline neural network in previous studies. For example, DeepNOG extended the encoding layer of DeepFam by representing amino acid characters into numerical vectors using a trainable model^24^. DeepPPF also adopted the basic architecture of DeepFam by using convolutional filters of various sizes and one-max pooling layer to model motif patterns^18^. To fully utilize the modeling capabilities of DeepFam, our work also adopted this architecture.

**Figure 1:**
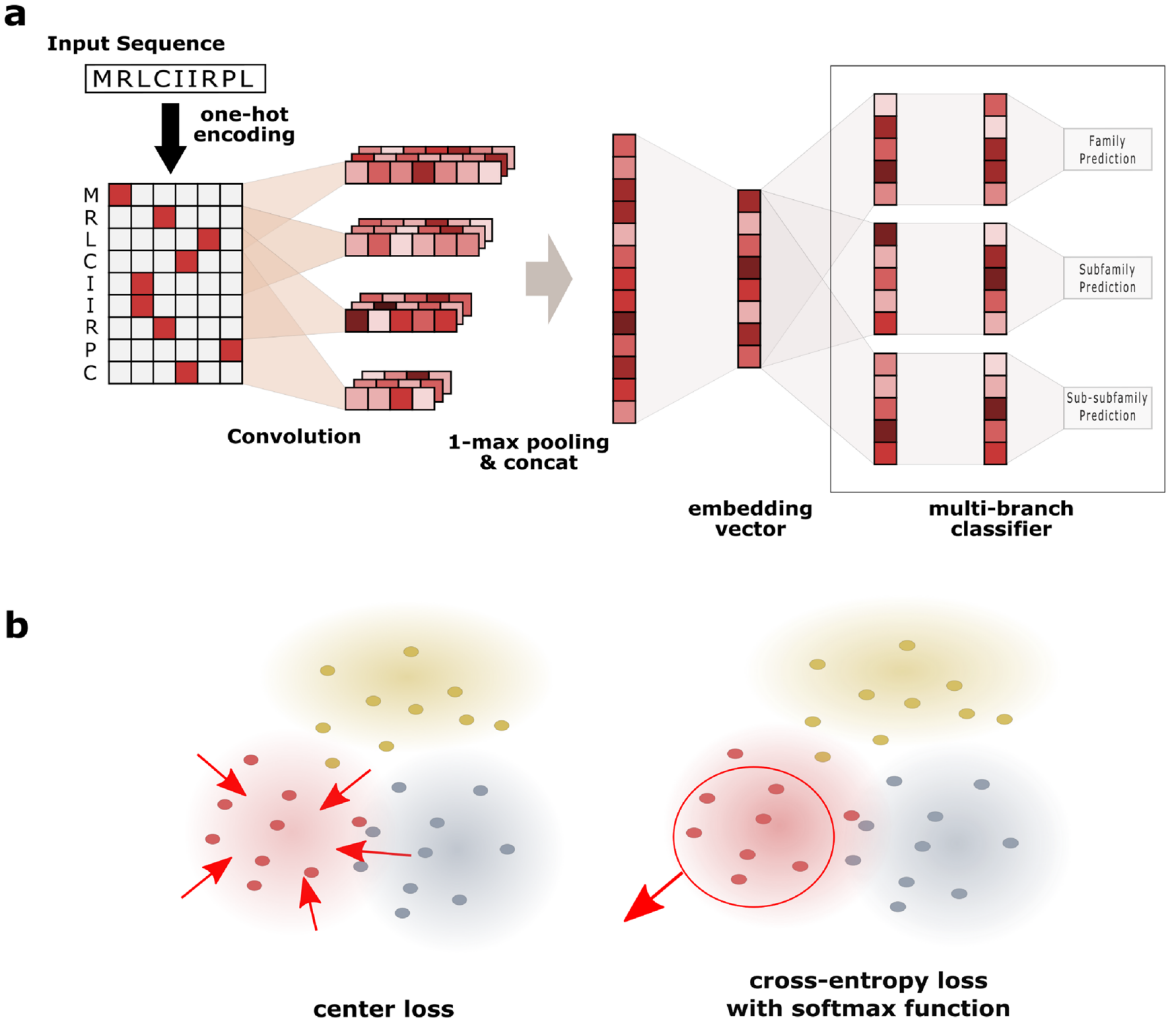
Overall figure of DeepHier. (**a**) Neural network architecture. (**b**) Loss function.

Rather than merely adopting the previous work, however, we introduce novel components on the architecture, Embedding layer and Multi-branch classifier, to effectively build a metric space. Overall architecture of DeepHier is illustrated in Fig. 1.

#### Feature extractor with CNN

In DeepFam, variable length convolutional filters were used with one-max pooling layer^17^. In such setting, output activation value from each convolutional filter encodes information on whether significant motif patterns are presented in the input sequences. This architecture was demonstrated to be successful in capturing motifs from biological sequences^16,17^. Especially, DeepFam was successful in detecting motifs of variable lengths. In this work, we also adopted this one-max pooling architecture since GPCR families are characterized by highly preserved sequence regions^25^. After applying one-max pooling, the resulting values in the convolutional layer is concatenated into an one-dimensional representation, which can be regarded as a list of existence values for learned motifs.

#### Embedding layer

A linear layer that comes after the convolutional layer is to project outputs from the convolutional layer onto a low-dimensional vector space. Projected vector will be used as an embedding vector of an input protein sequence. To simplify the explanation, we assume embedding vectors lie in a *d*-dimensional space. After the linear transformation follows an $L_2$ normalization operator on resulting vectors to keep the distances between embeddings from exploding. Thus, we restricted the vector representation of each sequence to lie in a *d*-dimensional hyper-sphere.

#### Multi-branch classifier

Three branches of Multilayer Perceptron (MLP) classifier are attached to the embedding layer. Techniques of using branches in a single neural network, with each branch dedicated to a domain-specific task, were proposed in Multi-Domain Network (MDNet)^26^ to construct shared features across the multiple domains. In our architecture, three branches correspond to each hierarchical level: class, family and subfamily level. Under this multi-branch architecture, gradients from these classifiers will guide embedding vectors to incorporate hierarchical information regarding GPCR protein family into a single representation.

### Loss function

In DeepHier, a loss function with respect to a metric space is introduced to construct meaningful distance relations between protein sequences. We first list the notations used in defining the loss terms in Table 1. In the table, $\mu _{y_i}$ is calculated as a mean vector of deep representations of sequences that corresponds to class $y_i$ proteins^21^ .

#### Cross-entropy loss with a softmax function

Cross-entropy loss with a softmax function is frequently used as a loss function for training classifiers. Softmax function is used to generate probability distribution of candidate labels from the output layer of the network. In many cases, softmax function is combined with cross-entropy loss in supervised setting to enforce classifiers to output higher probability on desired labels. This is effective for neural network models in learning separable representations between different classes. Likewise, we employed this function as a part of the loss to empower neural networks to learn separable features in input sequences for each label.

#### Center loss

Center loss^21^ was proposed to complement the cross-entropy loss with a softmax function. Although cross-entropy loss with a softmax function is practical enough to separate features between classes, it lacks ability to learn compact representations between data that is in the same class^21,22^. To address this, this additional loss term minimizes the distances among data points within a same class. Center loss can be stated in following form:

1 $$\begin{aligned} {L}_{C} = \sum _{i=1}^{n}{{\left\| {d(x_{i}) - \mu _{y_i}}\right\| }^2_2} \end{aligned}$$

With loss from this metric, parameters are updated to make representation of the input data get closer to the mean vector during training. Computer vision community have utilized this form of metric loss functions when there are larger number of classes. Especially, domains like face recognition problems were benefited from metric losses^21,27^. GPCR protein modeling is similar to these applications since we need to model relatively large number of classes (86 sub-subfamilies) with few number of data samples. Hence, we used center loss to train DeepHier.

In exploiting the above loss function, mean vectors should be updated simultaneously with parameters being updated. In the previous work^21^, mean vectors are calculated based on the images in mini-batch basis since considering vector representations of the whole dataset, generally comprising of 50K to 200M images for computer vision, is computationally exhaustive. However, the number of sequences in the training data is relatively small, total of 8222 GPCR proteins for our study. Therefore, we updated mean vectors of classes based on the feature vectors from the whole dataset.

**Table 1:** Notations related to loss terms.

*L*	Total loss to be used in training
$L_{S}$	Summed cross-entropy loss with a softmax function from three hierarchies
$L_{S_{i}}$	Cross-entropy loss with a softmax function from hierarchy level i where $i \in \{cls, fam, sub\}$
$L_{C}$	Summed center loss from three hierarchies
$L_{C_{i}}$	Center loss from hierarchy level i
$d(x_i)$	Embedding vector in the hidden layer for input sequence $x_i$
$\mu _{y_i}$	Class center of embedding vectors in deep feature space for class that input $x_i$ belongs to

#### Overall loss

Combining cross-entropy loss ${L}_{S}$ from the classifiers and center loss for each hierarchy, overall loss function can be stated in the following equation.

2 $$\begin{aligned} {L} = \sum _{i\in {S}}\omega _{S_i}{L}_{S_i} + \lambda _{C}\left( \sum _{i\in {S}}\omega _{C_i}{L}_{C_i}\right) \end{aligned}$$

where *S* denotes the set of hierarchy levels (S = {family, sub-family, sub-subfamily}) and loss function is stated as a weighted sum of losses from each hierarchy. To balance between center loss and cross-entropy loss with a softmax function, $\lambda _{C}$ was introduced as a weight for center loss^21^. Here, $\omega _{S_i}$ and $\omega _{c_i}$ represent the weight for cross-entropy loss with a softmax function, and center loss respectively.

### Training procedure

To effectively incorporate hierarchical information into a single embedding space, we designed a training procedure in three different phases. At each phase of training procedures, we devised DeepHier to focus on loss values from one level, from family-level to sub-subfamily level. In our setting, training phases are ordered following the hierarchical order, from family level to sub-subfamily level. This order ensures neural networks to firstly learn general representations that correspond to family level properties of GPCR families and then acquire fine-grained representations for sub-subfamily level.

### Analyzing distances in the embedding space

To assess the quality of distances in the proposed space, we evaluated how well the distances in the embedding space represent the hierarchical class structure of GPCR protein family. Embedding vectors from DeepHier were compared to following methods: (1) model with the same architecture as DeepHier but without using the center loss function (w/o Center Loss); (2) model with the same loss function and feature extractor as in DeepHier but without a multiple branches output layer (w/o Multiple Branch); (3) DeepFam^17^, a one-layer convolutional neural network with a one-max pooling operator that captures sequence motifs; (4) DeepPPF^18^, a novel convolutional neural network that trained encoding layers for amino acid characters; (5) Autoencoder, a convolutional autoencoder trained with reconstruction loss; (6) MLP classifier on a flattened one-hot encoding vector; and (7) K-mer frequency vectors (3-mer and 4-mer). For models based on neural networks, activations of neurons at the last hidden layer were used as representations for the sequences.

To be specific, clusters were compared to the class labels at family, subfamily and sub-subfamily levels using adjusted mutual information (AMI) and silhouette scores. In calculating the silhouette scores, real class labels from three hierarchical levels were used as groundtruth. Since silhouette score is related with intra-cluster and inter-cluster distances, silhouette scores under our scheme represent the consistency of distances in embedding space to the real class labels. Correspondence between the hierarchical clustering results and GPCR family labels was evaluated using AMI score. In estimating the score, we used the real label information as ground-truth label information to measure the quality of clustering results. Agglomerative clustering based on ward linkage, a hierarchical clustering algorithm where pairs of clusters with closest distances are merged together, were applied to the embedding vectors until the target number of clusters are obtained. In our experiment, we increased the target number of clusters from 2 to 100 with a step size of 3. AMI scores were measured between class labels and cluster results from each of the target cluster number. We provide detailed explanations on AMI and silhouette scores in the supplementary material.

### Analyzing the hierarchical structure in the embedding vectors

Relationship between proteins on the hierarchical class structure is an important aspect to be investigated in GPCR protein studies. We analyzed the overall distance relations between proteins in the embedding space compared to the real class labels. Afterwards, we further constructed a phylogenetic tree from the embedding vectors in order to demonstrate that phylogenetic analysis is possible with embedding vectors. Here, a phylogenetic tree was constructed using a neighbor-joining clustering method^28^.

### Discovering motifs from the embedding vectors

As a way of demonstrating the biological significance of the vectors, we performed motif discovery experiments on the deep feature vectors. In these experiments, MUSCLE, a multiple sequence alignment tool^29^, was used to detect conserved sequence patterns. Sets of reference proteins were selected from the hierarchical clustering results in the above section where target number of clusters were incremented. On the sequences in the designated clusters, we applied MUSCLE to figure out the highly preserved regions of that cluster. Afterwards, preserved regions from the alignment algorithm were compared to known motifs in the literatures to check if results are consistent with biological knowledge.

### Query search on embedding spaces

We conducted a sequence similarity query search on the protein family database as one of the downstream tasks on the embedding space from DeepHier. Distances in the low-dimensional embedding space have long been utilized for effective query search in the high dimensional data. In this setting, similarity between data points is defined in terms of distances in the low-dimensional space^30^. In a similar manner, since deep feature space was trained to express GPCR similarity in terms of metric distances, we directly utilize the euclidean distances between embedding vectors. We handled query search in terms of nearest points in the embedding space. In such setting, unlike conventional alignment-based algorithms for sequences, distance calculations on embedding space could be accomplished simply with numerical calculations. We compared the results with Basic Local Alignment Search Tool (BLAST), the *de facto* standard tool for sequence similarity search^31^. Since computation speed has been major limitations of alignment-based tools, we compared the execution time of each algorithm.

## Experimental setup

### Dataset preparation

The GPCR sequences were acquired from the BIAS-PROFS GPCR dataset^9^. In this dataset, sequence labels are annotated in three-level hierarchies, from the family level to the sub-subfamily level. In processing the dataset, classes with fewer than 10 sequences were removed, resulting in 86 sub-subfamilies and 8222 sequences. During the experiment for query search, sequences from the training dataset were utilized as a target database of approximately 6200 sequences. In contrast, query sequences were only retrieved from the test dataset.

### Sequence representation

To enable computations on deep learning models, one-hot encoding scheme was adopted for DeepHier, where every amino acid position was represented as a one-hot vector^32,33^. In addition, each sequence was padded with zeros to a fixed length because CNN requires input vectors of the same size. Thus, after converting a sequence into one-hot vectors, we padded each sequence to the length of 1000^17^. As a result of encoding, each sequence was converted into a vector of size $R^{1000 \times 20}$. Here, 20 is the alphabet size of amino acid characters.

### Hyperparameter setting

Hyperparameters used in the experiments are listed in Table 2. Parameters regarding convolutional layer were identical to those of DeepFam. However, in our work, we did not employ dropout operator since the embedding vectors need to be learned consistently while training. In addition, the dimension of embedding vectors needs to be set in advance. We selected the dimension of embedding vectors using validation dataset according to accuracies on sub-subfamily level classification. Weights for softmax and center loss terms are also set, separately for the family, subfamily and sub-subfamily phase.

**Table 2:** List of hyperparameters.

Hyperparameter	Value		
Kernel sizes	8, 12, 16, 20, 24, 28, 32, 36		
Number of filters	256 $\times$ 8		
Dimension of embedding vectors	30		
Number of hidden units in classifier	15		
Learning rate	0.001		
L2-regularizer on classifier	0.0005		
Weight on center loss ($\lambda _{C}$)	0.01	0.3	0.5
Weight for center loss on family level ($\omega _{C_{cls}}$)	0.8	0.1	0.1
Weight for center loss on subfamily level ($\omega _{C_{fam}}$)	0.15	0.8	0.15
Weight for center loss on sub-subfamily level ($\omega _{C_{sub}}$)	0.05	0.1	0.75
Weight for cross-entropy loss with a softmax function on family level ($\omega _{S_{cls}}$)	0.8	0.1	0.1
Weight for cross-entropy loss with a softmax function on subfamily level ($\omega _{S_{fam}}$)	0.15	0.8	0.25
Weight for cross-entropy loss with a softmax function on sub-subfamily level ($\omega _{S_{sub}}$)	0.05	0.1	0.65

### Implementation details

Neural network models were implemented with Python 3.6.8 and PyTorch 1.1.0^34^, an open-source deep learning library. Dataset was split into train, validation and test datasets with ratios of 0.8, 0.1 and 0.1 respectively for each subfamily class. We selected the best performance model on validation set and performances were measured in 10-fold cross-validation scheme. Only the embedding vectors from the test dataset were used for evaluation and visualization during experiments.

## Results

### Evaluation on embedding distances

#### Inspection on silhouette scores

**Figure 2:**
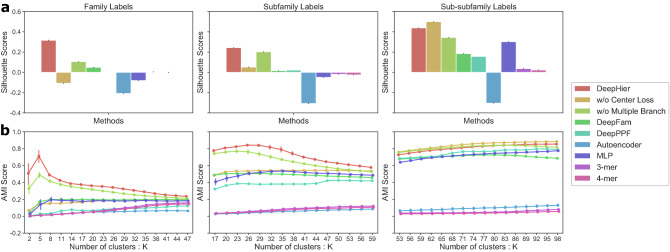
Evaluation on embedding distances based on clustering results. (**a**) Silhouette score of representation vectors regarding true class labels on three hierarchical levels. (**b**) Adjusted mutual information (AMI) score calculated for class label in each level versus *k* clusters yielded from hierarchical clustering algorithm.

Figure 2 contains the calculated silhouette score from each method. In the figure, only *DeepHier* and *DeepHier w/o multiple branch* are the model equipped with a center loss that constructs metric distances in deep feature space during training. The distinctive result from these models is that they show non-negative silhouette scores in three class levels, whereas vectors from other models present non-negative values only in one level and negative or near-zero scores in other levels. These demonstrate that center loss regarding metric space helped neural network to build effective distance relations for GPCR families.

Vectors from the Autoencoder and k-mer frequencies, that do not use any class information during training, show low scores in all levels. This result shows that label information was a powerful supervisor in training a model. Although DeepFam and DeepPPF achieved remarkable classification performances in their original works^17,18^, they did not show competitive scores in clustering qualities. These results indicate that center loss in DeepHier effectively constructed a meaningful embedding function compared to other models that do not address metric distances during training.

We now focus on comparison of two models that adopted center loss during parameter updates. Although the model without multiple branch shows positive scores for all three levels, our model shows significantly higher scores. In summary, the proposed two components for metric learning, multiple branches and center loss, have been successful in learning compact representations at all levels.

#### Inspection on AMI scores

Changes in AMI score for the number of clusters are illustrated in Fig. 2. AMI score is maximized when the number of resulting clusters from the algorithm gets closer to the real number of labels in that class level, 5 for family and 37 for subfamily. Interestingly, this can be interpreted as: hierarchical clustering results show best correspondence when a target cluster number is close to the actual number of class labels. This demonstrates that distance relations between embedding vectors match well with hierarchical class structure. Similar to results from the experiments of silhouette scores, some algorithms achieved comparable AMI scores in the sub-subfamily level clustering evaluation. However, DeepHier shows notably higher score in the family and subfamily levels. In fact, our model is the only method that generates the maximum AMI score higher than 0.7 for all class levels. This again supports that DeepHier was successful in incorporating information from all three class levels into a single unified embedding vector. In addition, comparison between results from DeepHier and DeepHier without center loss demonstrates that center loss in our approach makes our embedding more compactly represented.

### Analysis on the hierarchical structure

#### Overall distance structures in the embedding space

**Figure 3:**
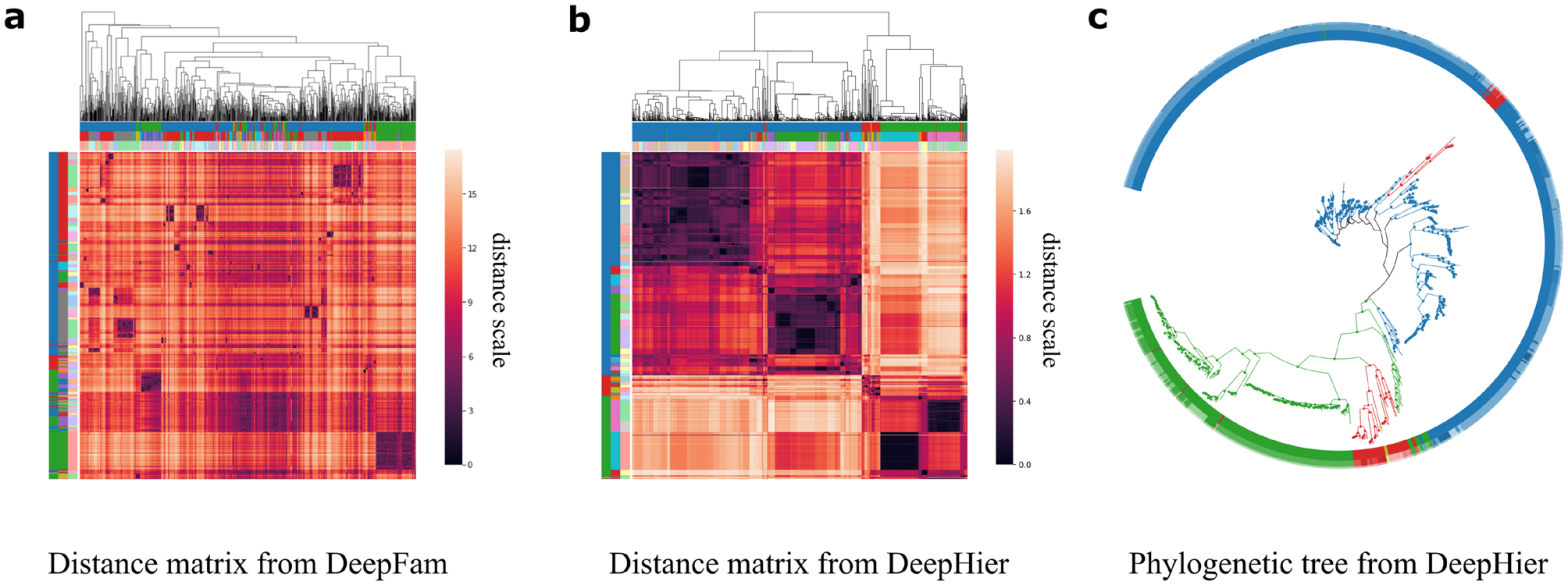
Embedding vectors on hierarchical structure, distance matrix and phylogenetic tree. (**a**) Distance matrix of embedding vectors from DeepFam. (**b**) Distance matrix of embedding vectors from DeepHier. (**c**) Phylogenetic tree reconstructed from DeepHier.

In Fig. 3, distance matrix was generated using pairwise euclidean distances between the embedding vectors. Protein sequence vectors were clustered using a hierarchical clustering algorithm, Unweighted pair group method with arithmetic mean (UPGMA)^35^. To visualize the hierarchical clustering result, columns were ordered based on the clustering results whereas rows were sorted according to family, subfamily, sub-subfamily class labels. Clustering results are demonstrated with dendrogram in the figure. Each of the three-line color bands on the columns and rows corresponds to the hierarchical class label of each sequence. Thus, correspondence between clustering results and true class information can be revealed by comparing three-line color bands on columns and rows.

It can be observed that hierarchical relations between sequences are apparent in the embedding space from DeepHier. Overall pairwise distances between the sequences in the heat map show that embedding vectors clearly captured the three distinctive distance relations among GPCR sequences, the brightest one ($1.6 \sim$), the middle-range one (1.2–1.6) and the darkest one ($\sim 0.5$). These distinctions in color level make separations between data points more clearly visible. Comparing boundary regions with true label information represented with color bands on columns and rows, color distinctions correctly correspond to the label information of sequences. For representations from DeepFam, although there exists local clusters of proteins, no hierarchical structure between distances is observed. Furthermore, hierarchical clustering results do not match with real class hierarchies in DeepFam. This demonstrates that even though DeepFam modeled sub-subfamily classes in GPCR protein families fairly well^17^, hierarchical structure of the proteins was not properly incorporated. In contrast, through employing center loss and multiple branches, DeepHier constructed distinctive hierarchical relations successfully with distances in the embedding space. We further provide t-SNE visualizations^36^ for the embedding vectors from DeepHierin the supplementary material.

#### Phylogenetic tree reconstruction

Phylogenetic tree from the embedding vectors is drawn in Fig. 3. In the tree, each family-level label is represented with a distinct color. A branch in the tree was assigned a color corresponding to the family when more than 70% of the leaves in the branch belong to a specific family. In addition, as we have done in generating the distance matrix, additional figure of overlaying three-layer color rings with colors corresponded to each class in the hierarchy. In the phylogenetic trees, sequences belonging to the same family labels were clustered in close positions. In the second and the third rings that represent subfamily and sub-subfamily respectively, sequences belonging to subfamily and sub-subfamily were also positioned in close locations in the tree. In sum, we created a single embedding space that can be used to construct a phylogenetic tree based on the single embedding space, grouping GPCR sequences closely at family, subfamily and sub-subfamily levels.

### Motif discovery

**Figure 4:**
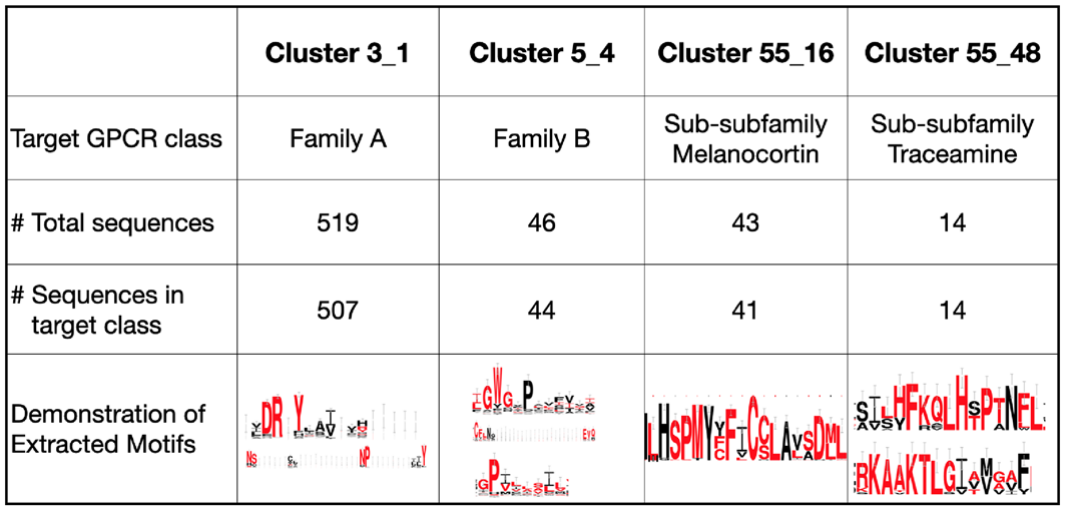
Discovered motif logos for each cluster on phylogenetic tree generated from hierarchical clustering results. Cluster information as well as information on the sequences included in the cluster is provided. Cluster 3-1 denotes that this cluster is first cluster from the hierarchical clustering when the target number of cluster is 3.

To visualize the discovered motifs, WebLogo, a web-based visualization tool^37^, was used in Fig. 4. From the clustering results with a target cluster number of three, conserved sequences of *DRY* and *NSY* were found to be distinctive features in the first cluster. This cluster had 519 sequences, of which 507 sequences belonged to Family A of GPCR protein family. In fact, the discovered motifs in these clusters are the most characterizable sequence features shown in family A or Rhodopsin GPCR family^1,38,39^. Unlike the first cluster, however, alignments on the other clusters does not reveal the conserved sequence of *DRY*. This corresponds to our knowledge that *DRY* and *NSY* are distinctive features for Rhodopsin-like GPCR proteins. Likewise, other significant motifs were found from other clusters too. On the fourth cluster among five clusters in the results, conserved sequences of *LIGWG*, *GPVLASLL* and *CFLxxEVQ* were discovered. These sequences belong to the conserved regions in the transmembrane structures of family B or Secretin receptor family of GPCR family^40^. Indeed, this cluster consists of 46 sequences where 44 of them belonged to family B GPCR proteins. At deeper hierarchical levels, *RKAAKTLG* and *FKQLHXPTN* were found to be conserved in the 48^th^ cluster among 55 clusters. These features are known to be the representative motifs in the Traceamine sub-subfamily that belongs to family A of GPCR proteins. This is consistent with the fact that all the sequences in the cluster are from Traceamine sub-subfamily.

### Query search speed on embedding space

We measured execution time of similarity search algorithm. Firstly, preparing database with DeepHier requires embedding the whole sequences in the training dataset through deep learning model, which is composed of nearly 6300 sequences. Second, time spent for searching through the database should be measured. Thus we compared execution time on database construction and database search. For DeepHier, database construction took approximately 12.7 s. In contrast, for BLAST, constructing database using *makeblastdb* command took 368 ms. For similarity search on embedding space, it took approximately 19 ms on average for single sequence when database is comprised of 6300 sequences. This includes time spent for representing query sequence in terms of embedding vector from the neural network. In contrast, BLAST took 266 ms on average.

**Figure 5:**
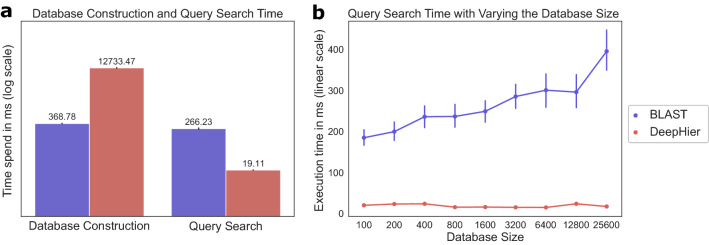
Execution time measured for query search algorithms. (**a**) Database construction time and query search time for the BIAS-PROFS GPCR dataset. (**b**) Time required for query search for different sizes of sequence database.

Constructing database in our approach is slower than that of conventional alignment-based approach, since DeepHier requires loading of neural network parameters and embedding of whole training sequences. However, once embeddings of the sequences in the database is constructed, we don’t need to repeat this process. Thus, we believe slow execution time in constructing database might not be a bottleneck in similarity search. For execution time spent on similarity search, our method only requires approximately 7.1.

To assess the scalability of similarity search when the size of the database grows, we measured the execution time with varying the size of database. BLAST shows linearly increasing execution time with the growth of database (Fig. 5). However, similarity search on the embedding vectors from DeepHier shows consistent execution time. We believe that since the embedding vectors lie in a low-dimensional vector space, distance can be easily calculated with matrix operation. Thus, we obtained scalability in similarity search. These results suggest that similarity search with our method can be a scalable alternative to BLAST for ever increasing size of biological sequences.

In addition to the execution time for query search, we estimated the accuracy of the search algorithm. DeepHier achieved classification accuracies of 0.985, 0.875, and 0.797 for family, subfamily, and sub-subfamily hierarchy. In contrast, query search with BLAST achieved accuracies of 0.989, 0.862, and 0.777, respectively. Except for family level classes, our method outperformed alignment-based algorithm in query search tasks. In sum, our method enables similarity search on biological sequences with comparable accuracy even without leveraging alignment-based computations.

## Discussion

We propose a deep learning based embedding function, DeepHier, that simultaneously learns significant protein features at three hierarchy levels of GPCR families. Sequence features in GPCR proteins are learned from the proposed model, which consists of three-branch classifiers and a novel loss term. The main advantage of simultaneous embedding of the hierarchies is that distances in the embedding space are directly correlated with hierarchical relations between proteins, which lies at the heart of GPCR studies. This is significant in that phylogenetic relationships of GPCR protein families can be modeled with the embedding vectors learnt from our deep learning model. In sum, we believe the proposed approach presented a novel and significant strategies for utilizing deep learning in analyzing GPCR protein sequences.

Until recently, biological sequence analyses relied heavily on alignment-based algorithms. However, recent deep learning works demonstrated that neural networks could be used to model biological sequences without loss of performances^17^. Nevertheless, a number of downstream tasks such as sequence query search or phylogenetic analysis were mostly done with multiple sequence alignments. In this regard, our work propose an embedding function where downstream analyses on biological sequences could be enabled without the expensive sequence alignment step. Throughout the experiments, we demonstrated that the embedding space effectively modeled GPCR family hierarchy into a single metric space. Furthermore, phylogenetic relations of the proteins were inferred with the proposed method. In addition, embedding space from DeepHier was utilized in sequence similarity search tasks for ever increasing GPCR databases. Experimental results in our work indicate that several downstream analysis on the protein sequences can be successfully accomplished with the embedding vectors generated from DeepHier.

In this study, we restricted the analysis on the embedding function to the GPCR sequences. However, as investigated in previous works^6,41^, discriminating GPCR proteins from non-GPCR proteins is also of great research interest. Additional classification hierarchy that determines whether input sequences are GPCR proteins or non-GPCR proteins could be added to the model. Previous machine learning work proposed a rejection option for the classifier based on the metric distances in the feature space^42^. In a similar regard, during the training, negative datasets or non-GPCR proteins could be fed into the model and we can train the model to embed vectors of GPCR proteins close in embedding space.

As a future work, information from the structural characteristics of GPCR receptor can be extensively incorporated into the training process in a similar manner to the proposed work. Since structural properties are crucial for identifying functions of the protein, such investigation might give opportunity to obtain more accurate modeling of GPCR proteins.

## Supplementary information

Supplementary Information.
