# Supplementary material for: Deep hierarchical embedding for simultaneous modeling of GPCR proteins in a unified metric space
Source: Sci Rep. 2021 May 5;11:9543. doi: 10.1038/s41598-021-88623-8 (PMC8100104; doi:10.1038/s41598-021-88623-8)
Supplement: Supplementary file 1 — Supplementary Information. [file 41598_2021_88623_MOESM1_ESM.pdf]

Supplementary Data to Deep Hierarchical Embedding for Simultaneous Modeling of GPCR Proteins in a Unified Metric Space

1 Performance metrics

To assess the quality of the embedding vectors, we performed quantitative evaluations on clusters that were constructed from the embedding vectors. Throughout the experiments, distances between embedding vectors were evaluated with regard to the actual class label, which is of hierarchical structure.

1.1 Silhouette score

Silhouette score measures the average value of the silhouette coefficients for each data point. Silhouette coefficient is widely used to assess the relative quality of clusters by evaluating the tightness and separability of data points given clustering labels[1]. This metric is to compare intra-cluster distances and inter-cluster distances. Silhouette coefficient for a data point i is calculated as follows:

$$s(i) = \frac{b(i) - a(i)}{max\{a(i), b(i)\}} \tag{1}$$

In the above equation, $a(i)$ denotes the mean distance between a given sample and other samples in the same cluster and $b(i)$ is the mean distance between the sample and the data points in the nearest cluster. Silhouette score can be calculated by taking an average on the above silhouette coefficients, resulting in values ranging between -1 and 1. In general, high silhouette score indicates that representations of data points correspond well to the class structure. For example, if intra-cluster data points are closely placed compared to data points of other clusters, silhouette score will be positive and close to one, indicating that the data points match well with the cluster membership. Otherwise, silhouette score of negative value implies the poor correspondence between data points and clustering results.

1.2 Adjusted mutual information score (AMI)

AMI is used to evaluate the correspondence between two clustering results[2]. This metric measures the quality of clustering by comparing the clustering result to the ground-truth class labels. This score utilizes information theoretic perspectives to compare results generated by clustering algorithms. For given two set of clusters, U and V, AMI score is defined as follows:

$$\mathrm{AMI}(U,V) = \frac{\mathrm{I}(U,V) - \mathrm{E}\{\mathrm{I}(U,V)\}}{max\{\mathrm{H}(U), \mathrm{H}(V)\} - \mathrm{E}\{I(U,V)\}} \tag{2}$$

In the above equation, I(U, V) is the mutual information between two results. H( ) is the information entropy measured for each cluster result. In AMI score, additional term E{I(U, V)} is introduced to compensate for agreements between two clusters arouse by chance. This index calculates the proportion of information shared between two clustering results.

2 t-SNE visualization of embedding vectors

2.1 Embedding vectors of the proposed method

In Figure 1, we visualized the distribution of embedding vectors. Upper left figure shows embedding vectors across different family and upper right figure colors embedding vectors following the subfamily labels for family A proteins. Two figures in the bottom displays distribution of vectors on sub-subfamily scale. In the figure, data points of same labels form distinctive clusters at all levels. This result suggests that the proposed method effectively represent characteristics of proteins at different hierarchical levels into a unified vectors.

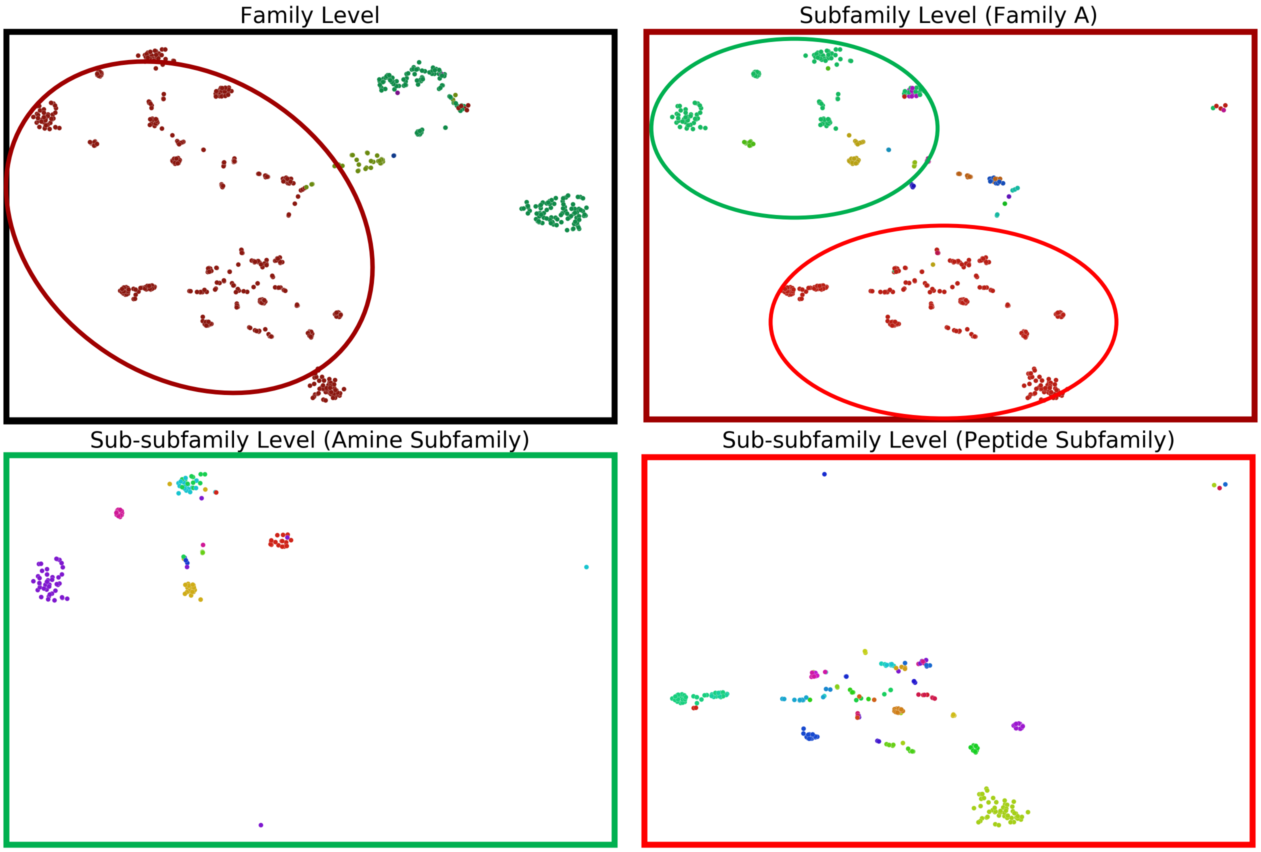

Figure 1. Visualization of the embedding vectors from the proposed method. t-SNE plot of the embedding vectors of GPCR families, subfamilies in GPCR family A and sub-subfamilies in Amine and Peptide subfamilies are illustrated.

2.2 Embedding vectors of DeepFam

In Figure 2, t-SNE plot of embedding vectors from DeepFam is provided. As shown in the Figure, embedding vectors are clustered relevant well for sub-subfamily level classes, which is consistent with the results reported in the paper. However, overall structure of clusters does not show good accordance with the class information at family and subfamily level.

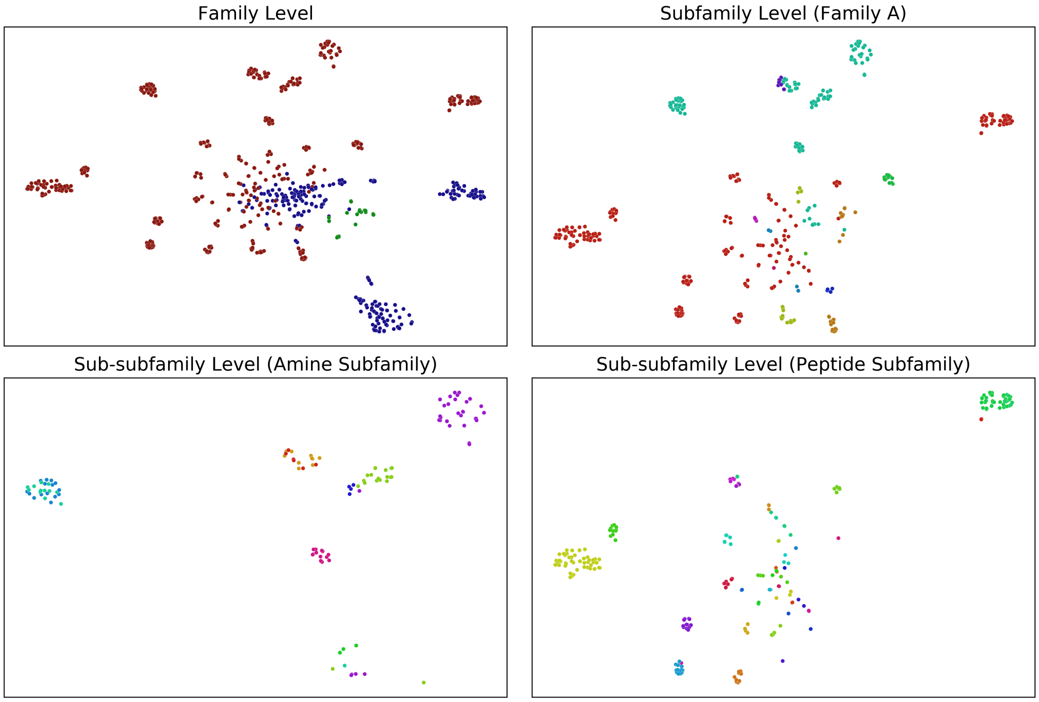

Figure 2. Visualization of the embedding vectors from DeepFam. t-SNE plot of the embedding vectors of GPCR families, subfamilies in GPCR family A and sub-subfamilies in Amine and Peptide subfamilies are illustrated.

3 Analyzing capsule network for learning hierarchical features

Recently, Capsule Network, a novel neural network architecture, was proposed to model hierarchical relationships between features[3]. Although the proposed work differs from capsule network in that hierarchical class structure was modeled in our study, we believe our work can be extended in the future work by relating hierarchical feature representation in capsule network with hierarchical class structure. Furthermore, as the future work, information from the structural characteristics of GPCR receptor can be extensively incorporated into the training process of neural network. Since structural property is crucial in identifying the functions of the protein, such investigation might give opportunity to obtain more accurate modeling of GPCR proteins.

References

1. Rousseeuw, P. J. Silhouettes: a graphical aid to the interpretation and validation of cluster analysis. *J. computational applied mathematics* **20**, 53–65 (1987).
2. Vinh, N. X., Epps, J. & Bailey, J. Information theoretic measures for clusterings comparison: Variants, properties, normalization and correction for chance. *J. Mach. Learn. Res.* **11**, 2837–2854 (2010).
3. Sabour, S., Frosst, N. & Hinton, G. E. Dynamic routing between capsules. In *Advances in neural information processing systems*, 3856–3866 (2017).
